# Effect of a mobile app on breastfeeding among cesarean deliveries: A randomized controlled trial

**DOI:** 10.1093/eurpub/ckac129.571

**Published:** 2022-10-25

**Authors:** TT Duong Doan, C Binns, A Lee, N Minh Pham, T Phuong Hoa Dinh, Y Zhao, TT Ha Bui

**Affiliations:** 1 Population and Reproductive Health, Hanoi University of Public Health, Hanoi, Vietnam; 2 School of Population Health, Curtin University, Perth, Australia; 3 Thai Nguyen University of Medicine and Pharmacy, Thai Nguyen, Vietnam

## Abstract

**Aim:**

Cesarean section has negative impacts on breastfeeding rates. This study is to evaluate the effect of a mobile application on breastfeeding outcomes among mothers who had cesarean section using a randomized control trial in Vietnam in 2020 - 2022.

**Methods:**

A triple-blinded randomized trial of a mobile application was conducted. The mobile application was tailored to Vietnamese culture with two separate versions for the intervention and the control group. The intervention version auto-generated three messages per week and linked the information in the library content to improve breastfeeding while the control version sent messages on maternal and child health care. Pregnant mothers were recruited during their antenatal visits and randomly assigned to two groups. Outcomes of interest included early initiation of breastfeeding and exclusive breastfeeding rates.

**Results:**

A total of 275 mothers in the control and 293 in the intervention group who had undergone a cesarean section were included in the analyses. Significant increases were observed for early initiated breastfeeding within two hours (aOR= 1.51, 95%CI: 1.01 to 2.27) and exclusive breastfeeding during hospital stay (aOR=1.59, 95%CI: 1.02 to 2.49).

**Conclusions:**

Our results support the use of a theory-based design mobile phone application as a part of a promising intervention to improve breastfeeding outcomes.

**Key messages:**

• A mobile phone application could be a widely accessible, acceptable, and effective intervention to improve breastfeeding outcomes.

• To improve exclusive breastfeeding rates, comprehensive interventions at different levels of sociocultural and market contexts, settings, and individuals are needed.

